# Nitric Oxide Plays a Central Role in Water Stress-Induced Tanshinone Production in *Salvia miltiorrhiza* Hairy Roots

**DOI:** 10.3390/molecules20057574

**Published:** 2015-04-24

**Authors:** Xuhong Du, Chenlu Zhang, Wanli Guo, Weibo Jin, Zongsuo Liang, Xijun Yan, Zhixin Guo, Yan Liu, Dongfeng Yang

**Affiliations:** 1 College of Life Sciences, Zhejiang Sci-Tech University, Hangzhou 310018, China; E-Mails: ydf807@163.com (X.D.); gwl1016@aliyun.com (W.G.); jwb@zstu.edu.cn (W.J.); 2 College of Biological Science & Engineering, Shaanxi University of Technology, Hanzhong 723000, China; E-Mail: chenluzhang@126.com; 3 Tasly R&D Institute, Tasly Holding Group Co. Ltd, Tianjin 300410, China; E-Mails: yxj@tasly.com (X.Y.); guozx@tasly.com (Z.G.); 4 Tianjin Tasly Modern TCM Resources Co., Ltd., Tianjin 300402, China; E-Mail: lyzc596392@163.com

**Keywords:** nitric oxide, tanshinone, *Salvia miltiorrhiza*, polyethylene glycol, abscisic acid

## Abstract

Nitric oxide (NO), a well-known signaling molecule plays an important role in abiotic and biotic stress-induced production of plant secondary metabolites. In this study, roles of NO in water stress-induced tanshinone production in *Salvia miltiorrhiza* hairy roots were investigated. The results showed that accumulations of four tanshinone compounds in *S. miltiorrhiza* hairy roots were significantly stimulated by sodium nitroprusside (SNP, a NO donor) at 100 μM. Effects of SNP were just partially arrested by the mevalonate (MVA) pathway inhibitor (mevinolin), but were completely inhibited by the 2-C-methyl-d-erythritol-4-phosphate pathway (MEP) inhibitor (fosmidomycin). The increase of tanshinone accumulation and the up-regulation of *HMGR* and *DXR* expression by PEG and ABA treatments were partially inhibited by an inhibitor of NO biosynthesis (*N*^ω^-nitro-L-arginine methyl ester (L-NAME)) and a NO scavenger (2-(4-Carboxyphenyl)-4,4,5,5-tetramethylimidazoline-1-oxyl-3-oxide (c-PTIO)). Simultaneously, NO generation in the hairy roots was triggered by PEG and ABA, and the effects were also arrested by c-PTIO and L-NAME. These results indicated that NO signaling probably plays a central role in water stress-induced tanshinone production in *S. miltiorrhiza* hairy roots. SNP mainly stimulated the MEP pathway to increase tanshinone accumulation.

## 1. Introduction

Secondary metabolites, important sources for pharmaceuticals, food additives, flavors, and other industrial materials, are an important focus of crop breeding and metabolic engineering. Although secondary metabolites are generally nonessential for the maintenance of fundamental life processes, they play important roles in the interaction between plants and their environments [1]. Accumulations of secondary metabolites are widely stimulated by various stresses [2]. Signal transduction is a necessary cellar process for secondary metabolite accumulation in plants subjected to stresses. A better understanding of signal transduction involved in secondary metabolism will not only improve our knowledge of fundamental biology, but also lead to the production of useful secondary metabolites. Drought, a globe problem, has been shown to increase the amounts of secondary metabolites, including terpenoids [3]. Nitric oxide (NO) and abscisic acid (ABA) are well-known signal molecules induced in plants by various stresses, including water stress. ABA is defined as a stress plant hormone because of its rapid accumulation in response to water stress, and it plays a major role in the regulation of plant growth, development and tolerance under stress [4]. It has been reported that ABA can induce the accumulation of terpenoids in plants [5]. NO has been intensively studied to elucidate the role of this enigmatic signaling molecule in response to abiotic and biotic stresses such as drought stress and heat stress [6]. ***N***^ω^-nitro-L-arginine methyl ester (l-NAME) is a specific inhibitor of NO synthase [7], and 2-(4-Carboxyphenyl)-4,4,5,5-tetramethylimidazoline-1-oxyl-3-oxide (c-PTIO) is a scavenger of NO in cells. Drought stress also induces generation of NO [8]. The effects of NO on terpenoid production have been widely observed [9]. In maize leaves, NO mediates brassinosteroid-induced ABA biosynthesis involved in oxidative stress tolerance [7]. There is also evidence that NO accumulation in guard cells in response to osmotic stress mediates ABA-induced stomatal closure [10].

*Salvia miltiorrhiza*, one of the most popular traditional Chinese medicines, has been widely used in prevention and treatment of coronary heart disease [11]. Tanshinones, a group of diterpenoids, are the main biologically active ingredients in *S. miltiorrhiza*. Tanshinones are biosynthesized via the mevalonate (MVA) pathway in the cytosol and the 2-C-methyl-d-erythritol-4-phosphate (MEP) pathway in the plastids [12]. 3-Hydroxy-3-methylglutarylcoenzyme A reductase (HMGR) is the rate-limiting enzyme in the MVA pathway, and mevinolin (MEV) is a highly specific inhibitor of HMGR [13]. 1-Deoxy-d-xylulose 5-phosphate reductoisomerase (DXR) is the second enzyme in the MEP pathway, and fosmidomycin (FOS) is a specific inhibitor of DXR. Previous work revealed that tanshinone accumulations in *S. miltiorrhiza* were mainly from the MEP pathway, but could partially depend on the crosstalk between the two pathways [12,14]. Our previous work revealed that tanshinone accumulation in *S. miltiorrhiza* was stimulated by water stress in pot experiments [15]. In *S. miltiorrhiza* hairy roots, both polyethylene glycol (PEG) and ABA could enhance tanshinone production via the MEP pathway [16]. Application of sodium nitroprusside (SNP), a donor of NO, also resulted in a significant increase of tanshinone production [17]. However, the role(s) of NO in water stress-induced tanshinone is still unclear. In this work, we revealed that SNP mainly stimulated the MEP pathway to increase tanshinone accumulation. NO signaling probably plays a central role in water stress-induced tanshinone production in *S. miltiorrhiza* hairy roots.

## 2. Results

### 2.1. Effects of SNP on Tanshinone Production in S. miltiorrhiza Hairy Roots

**Figure 1:**
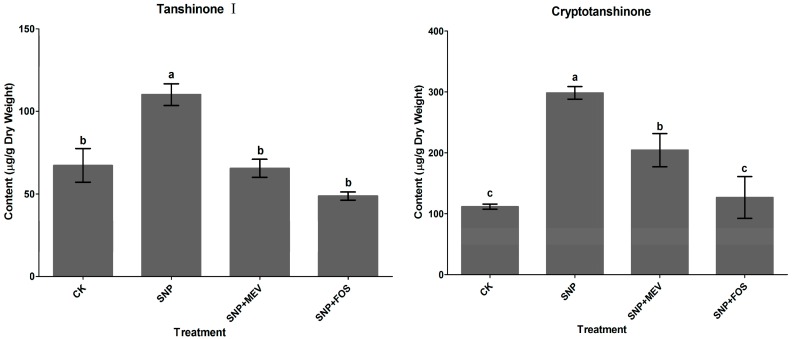

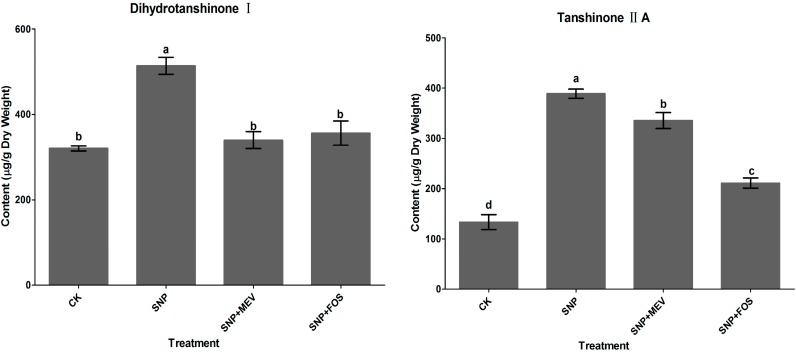
Effects of mevinolin (10 µM) and fosmidomycin (150 µM) on SNP-induced tanshinone production in *S. miltiorrhiza* hairy roots. CK, the control; SNP, sodium nitroprusside, 100 µM. Different letters (a, b, c and d) indicate significant difference at *p* ≤ 0.05. Means ± standard deviation (S.D.) (*n* = 3) are shown.

As shown in Figure 1, the accumulation of four tanshinones, including tanshinone IIA, cryptotanshinone, dihydrotanshinone I and tanshinone I was stimulated by 100 μM SNP, and reached to 388.8, 298.4, 110.1 and 514.0 μg·g^−1^ respectively. To identify the specific pathway responsible for tanshinone biosynthesis, effects of the MVA and the MEP pathway blockers on SNP-induced tanshinone production were investigated. Figure 1 showed that increases of four tanshinones contents induced by SNP were significantly reduced by mevinolin and fosmidomycin. Contents of tanshinone I and dihydrotanshinone I were decreased to the control level by both mevinolin and fosmidomycin. Cryptotanshinone accumulation was partially inhibited by mevinolin (from 298.4 to 204.3 μg·g^−1^) and completely arrested by fosmidomycin (from 298.4 to 126.5 μg·g^−1^). Tanshinone IIA content was partially depressed by both mevinolin and fosmidomycin, and it was 335.5 μg·g^−1^ in SNP + MEV treatment and 211.0 μg·g^−1^ in SNP + FOS treatment. It indicated that both the MVA and the MEP pathways are responsible for SNP-induced accumulation of tanshinone I and dihydrotanshinone I, but the MEP pathway contributes mainly for cryptotanshinone and tanshinone IIA production. Thus separate regulation mechanisms were observed for different tanshinone components by SNP in our study.

### 2.2. Effects of c-PTIO and l-NAME on Water Stress-Induced Tanshinone Production

**Figure 2:**
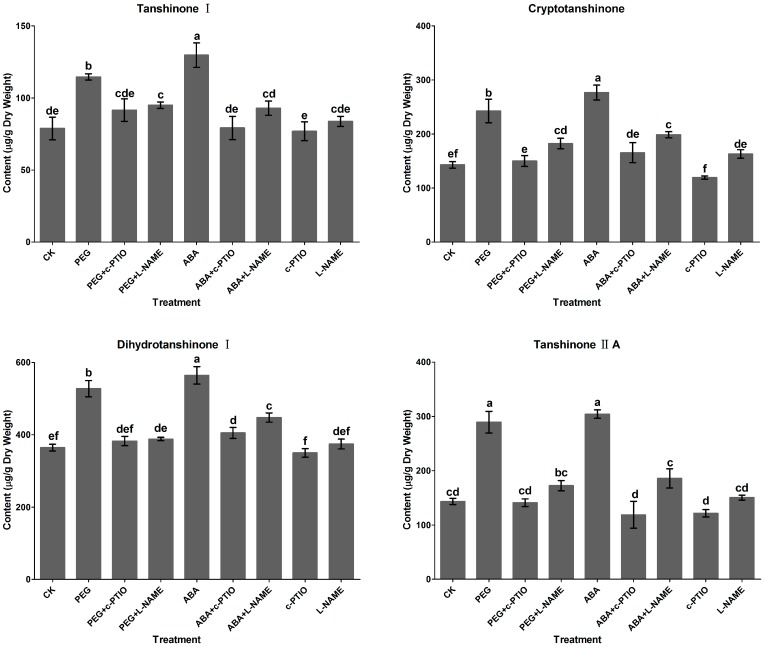
Effects of l-NAME (200 µM) and c-PTIO (200 µM) on PEG and ABA-induced tanshinone production in *S. miltiorrhiza* hairy roots. CK, the control; PEG, polyethylene glycol, 2% (W/W); ABA, abscisic acid, 200 µM; l-NAME: *N*^ω^-nitro-L-arginine methyl ester, 200 µM; c-PTIO, 2-(4-Carboxyphenyl)-4,4,5,5-tetramethylimidazoline-1-oxyl-3-oxide, 200 µM. Different letters (a, b, c, d, e and f) indicate significant difference at *p* ≤ 0.05. Means ± standard deviation (S.D.) (*n* = 3) are shown.

To investigate effects of water stress on tanshinone production in *S. miltiorrhiza* hairy roots, 2% PEG was added into the culture. The results showed that tanshinone production was significantly improved by 2% PEG treatment (Figure 2). Contents of tanshinone I, cryptotanshinone, dihydrotanshinone I and tanshinone IIA in PEG treatment increased by 70,117, 45 and 42% over the control, and reached to 114.6, 242.6, 464.4 and 189.3 μg·g^−1^, respectively. To clarify the role of NO in PEG-induced tanshinone production, c-PTIO and l-NAME were employed in our experiments. 200 µM c-PTIO and 200 µM l-NAME were added into the culture together with 2% PEG to inhibit NO biosynthesis, respectively. Figure 2 showed that the increase of four tanshinones contents were significantly inhibited by c-PTIO and l-NAME. Neither c-PTIO nor l-NAME alone could influence tanshinone accumulation. This demonstrated that PEG-induced tanshinone production was probably NO-dependent.

ABA (200 μM) was separately added into the culture to analyze its effects on tanshinone production. As shown in Figure 2, contents of tanshinone I, cryptotanshinone, dihydrotanshinone I and tanshinone IIA in hairy roots were significantly enhanced by ABA treatment, increasing by 0.9, 1.5, 0.6 and 0.5-fold over the control, and reaching to 124.7, 276.8, 527.7 and 204.1 μg·g^−1^, respectively. The increase of tanshinone contents induced by ABA was completely reduced by c-PTIO and l-NAME to the control level, which meant that NO probably mediated ABA-induced tanshinones production.

### 2.3. Generation of Nitric Oxide Induced by Water Stress

**Figure 3:**
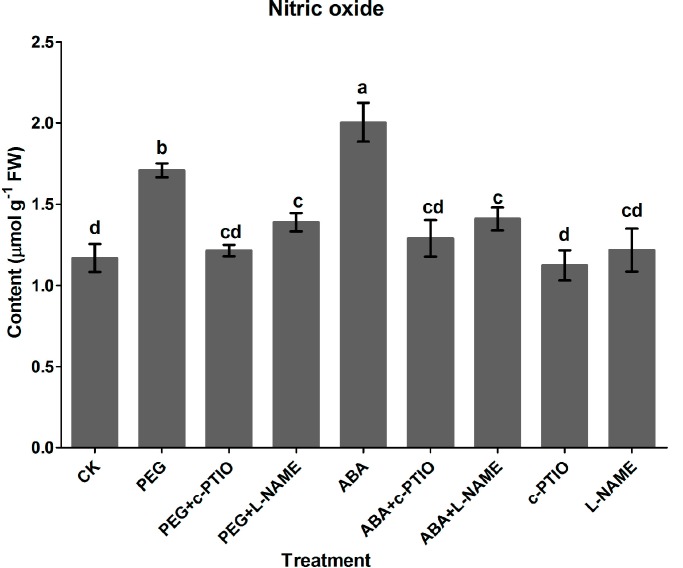
Generation of NO in *S. miltiorrhiza* hairy roots induced by 2% PEG and 200 µM ABA. CK, the control; PEG, polyethylene glycol; ABA, abscisic acid; l-NAME: *N*^ω^-nitro-L-arginine methyl ester, 200 µM; c-PTIO, 2-(4-Carboxyphenyl)-4,4,5,5-tetramethylimidazoline-1-oxyl-3-oxide (200 µM). Different letters (a, b, c and d) indicate significant difference at *p* ≤ 0.05. Means ± standard deviation (S.D.) (*n* = 3) are shown.

To elucidate further roles of NO in PEG- and ABA-induced tanshinone production, NO production in *S. miltiorrhiza* hairy roots was determined. As shown in Figure 3, NO productions were significantly stimulated by PEG and ABA treatments (1.5 and 1.7-fold of the control) and reached to 1.7 and 2.0 µmol·g^−1^·FW. Increases of NO accumulation induced by PEG and ABA were largely arrested by c-PTIO and l-NAME, and reduced to the control level. NO content was only slightly influenced by either c-PTIO or l-NAME. This further confirmed our conclusion that PEG and ABA-induced tanshinone production depended on NO signaling.

**Figure 4:**
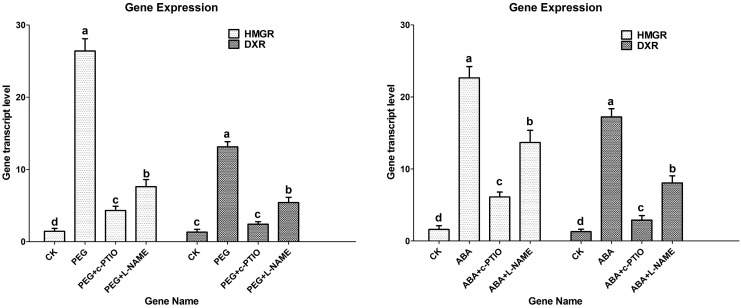
Expressions of *HMGR* and *DXR* induced by 2% PEG and 200 µM ABA. *HMGR*, hydroxymethyl glutaryl-coenzyme A reductase gene; *DXR*, 1-deoxy-D-xylulose 5-phosphate reductoisomerase gene; CK, the control; PEG, polyethylene glycol; ABA, abscisic acid; l-NAME: *N*^ω^-nitro-L-arginine methyl ester, 200 µM; c-PTIO, 2-(4-Carboxyphenyl)-4,4,5,5-tetramethylimidazoline-1-oxyl-3-oxide, 200 µM. Different letters (a, b, c and d) indicate significant difference at *p* ≤ 0.05. Means ± standard deviation (S.D.) (*n* = 3) are shown.

### 2.4. Effects of Water Stress on Expressions of HMGR and DXR

In the MAV and the MEP pathways, tens of enzymes are involved in the production of terpenoids. For example, HMGR is involved in the MVA pathway and DXR is involved in the MEP pathway. In *S. miltiorrhiza*, genes encoding HMGR [18] and DXR [19,20] have been cloned. To further elucidate the role(s) of NO in water stress-induced tanshinone accumulation, expressions of *HMGR* and *DXR* were detected by RT-PCR. Figure 4 showed that expressions of *HMGR* and *DXR* were sharply up-regulated by PEG and ABA. Transcript levels of *HMGR* and *DXR* increased by 26 and 13-fold over the control in PEG treatment, 23 and 17-fold in ABA treatment. Simultaneously, the up-regulations of *HMGR* and *DXR* induced by PEG and ABA were largely arrested by c-PTIO and l-NAME. These provided us the evidence at transcript level that PEG and ABA-induced tanshinone production depended on NO signaling.

## 3. Discussion

NO as a biological signaling molecule has been observed to be induced by various environmental stresses and elicitors, such as drought [8], salt [21], fungal elicitors [22], ABA [10], SNP [8] and MJ [23]. Some studies have shown that NO is involved in secondary metabolism. In *Catharanthus roseus* cell suspension cultures, SNP triggered terpenoid indole alkaloid biosynthesis [24]. In *Taxus* cells, NO plays a signal role in the ultrasound and cerebroside-induced taxol production [9]. In our previous work, we found that tanshinone production was significantly increased by SNP [17]. The results held true in this work. Tanshinone production in *S. miltiorrhiza* hairy roots was significantly stimulated by 100 μM SNP. Simultaneously, expressions of *HMGR* and *DXR* were both up-regulated by SNP. This work confirmed that NO was an effective elicitor for tanshinone production. Our previous work has revealed that the MEP pathway plays a major role in tanshinone production under PEG and ABA treatment [13,17]. In the present experiments, we found that effects of SNP were just partially arrested by mevinolin, but completely inhibited by fosmidomycin. The results indicated that both the MVA and the MEP pathways were induced by SNP, but the MEP pathway probably contributed mainly to SNP-induced tanshinone production.

Roles of NO in water stress-induced responses of plants have been widely reported. In *Vicia faba*, NO production was largely promoted by PEG and was completely inhibited by l-NAME [8]. It was believed that NO signaling mediated drought-induced ABA biosynthesis [7]. There was also evidence that ABA enhanced NO biosynthesis in stomatal guard cells [10] and NO induced ABA accumulation in root tips of wheat seedlings [25]. Our previous work has revealed that contents of tanshinone components in *S. miltiorrhiza* were significantly improved by drought stress in pot experiments [15]. PEG and ABA can enhance tanshinone production in *S. miltiorrhiza* hairy roots [16,26]. These previous observations were confirmed in the present work. Tanshinone production and expressions of *HMGR* and *DXR* in *S. miltiorrhiza* hairy roots were significantly induced by PEG and ABA. However, the role(s) of NO in water-induced tanshinone production are still unclear. The role of NO in terpenoid production has been widely observed [9]. In *Taxus* cells, a rapid production of NO was induced by MJ and it was shown that NO signaling is involved in MJ-induced taxol production [23]. More and more studies have revealed the interactions of NO with ABA signaling [7,25]. The induction of ABA by drought was strongly blocked by pretreating the root tips with nitric oxide synthase inhibitor, and SNP can also induce ABA accumulation in root tips of wheat seedlings [25]. We found that the increase of tanshinone accumulations and up-regulation of *HMGR* and *DXR* expressions by PEG and ABA treatments were inhibited by l-NAME and c-PTIO. Simultaneously, NO generations triggered by PEG and ABA were also arrested by l-NAME and c-PTIO. The results demonstrated that NO probably mediated PEG and ABA-induced tanshinone production.

## 4. Experimental Section

### 4.1. Hairy Root Culture and Treatment

Leaves of *S. miltiorrhiza* plantlets were infected by *Agrobacterium rhizogenes* bacterium (ATCC15834) to obtain hairy roots. Hairy roots (0.3 g fresh weight) were cultivated in a 250-mL shake flask containing 50 mL of the hormone-free liquid 6,7-V medium on orbital shaker. The shaker was set at 110 rpm and 25 °C in the dark.

Stock solutions of ABA (Wolsen, Xi’an, China), sodium nitroprusside (Sigma, St. Louis, MO, USA), ***N***^ω^-nitro-l-arginine methyl ester hydrochloride (Sigma), c-PTIO (Amresco, Cleveland, OH, USA) and fosmidomycin sodium salt (Santa Cruz Biotechnology, Dallas, TX, USA) were prepared in distilled water, then sterilized by filtration (0.22 membrane µm). Mevinolin (Sigma, St. Louis, MO, USA) was converted to the water-soluble sodium salt as described [27]. Polyethylene glycol 6000 (Merck, Darmstadt, Germany) was sterilized by autoclaving at 121 °C for 30 min. Final concentrations of 100 µM SNP, 10 µM mevinolin, 150 µM fosmidomycin, 200 µM l-NAME, 200 µM c-PTIO, 2% PEG and 200 µM ABA were used for treatments. The treatments were conducted on day 18th post inoculation of the hairy root culture. Hairy roots were harvested at day 6 after treatments and then dried at 45 °C. All treatments were performed in triplicate.

### 4.2. Determination of Nitric Oxide

Content of nitric oxidein hairy roots was determined by nitric oxide determination kit (Nanjing Jiancheng, Nanjing, Jiangshu, China) according to manufacturer’s direction. Because NO has a short half-life (2–30 s), it is quickly oxidized to nitrite (NO_2_^−^) and nitrate (NO_3_^−^) in the tissue. NO_3_^−^ can be converted into NO_2_^−^ by nitrate reductase, and NO_2_^−^ can be detected spectrophotometrically using the Griess Reaction. This kit is sensitive, stable and simple to use, with a major advantage of measuring the total amount of NO_3_^−^ and NO_2_^−^ through nitrate reductase.

### 4.3. Extraction and HPLC Analysis of Tanshinone

The powder (0.1 g) of dried roots was extracted ultrasonically with methanol-water solution (7:3, 2 mL) for 45 min. The extracts were centrifuged at 10,000 rpm for 15 min and then filtered through a 0.45-µm Millipore filter. HPLC analysis of tanshinone IIA, cryptotanshinone, dihydrotanshinone I and tanshinone I was performed as per our previous work [12]. HPLC was performed with a Waters (Milford, MA, USA) binary pump and photodiode array detector (DAD). The column was a Waters SunFire C_18_ (250 mm × 4.6 mm, 5 µm). Data were acquired and processed by Empower2 software (Milford, MA, USA). Separation was achieved by a linear gradient with solvent-A (acetonitrile) and solvent-B (water). The gradient was: t = 0 min, 40% A; t = 5 min, 60% A; t = 20 min, 60% A; t = 23 min, 80% A; t = 25 min, 100% A. The flow rate was 1 mL·min^−1^, the column temperature was 30 °C, and the injection volume was 20 µL. The effluent was monitored between 200 and 400 nm by DAD.

### 4.4. Quantitative Real Time PCR

The fresh hairy roots were homogenized in liquid nitrogen to a fine power. The total RNA was extracted by RNAiso^TM^ Plus (Takara, Dalian, China). Then, the first strand cDNA was synthesized from 500 ng total RNA with PrimeScript^®^ RT reagent Kit (Takara). Using the cDNA as template, transcript levels of *HMGR* and *DXR* were detected by a Bio-Rad CFX96 system (Bio-Rad, Hercules, CA, USA) with Brilliant II SYBR^®^ Green QPCR Master Mix (Agilent, Santa Clara, CA, USA). *β-actin* was the reference gene. The primers were designed by the software Primer-Premier 5.0 (Premier Biosoft International, Palo Alto, CA, USA) (Table 1).

**Table 1:** The primers of genes in RT-PCR.

Name	Sequence	Gene Bank ID
HMGR-F	5'-GCAACATCGTCTCCGCCGTCTACA-3'	FJ747636
HMGR-R	5'-GATGGTGGCCAGCAGCCTGGAGTT-3'	FJ747636
DXR-F	5'-CATGCGTTTGCCTATTCTGTAC-3'	DQ991431
DXR-R	5'-ACTAAGAACTCCGGTCATGGTG-3'	DQ991431
β-actin-F	5'-AGGAACCACCGATCCAGACA-3'	DQ243702.1
β-actin-R	5'-GGTGCCCTGAGGTCCTGTT-3'	DQ243702.1

### 4.5. Statistical Analysis

One-way analysis of variance (ANOVA) with Duncan’s multiple-range test was performed using Data Processing System (DPS) for Windows (Hangzhou, Zhejiang, China). The difference among different values was considered to be statistically significant when *p* ≤ 0.05.

## 5. Conclusions

In conclusion, we have presented evidence that both the MVA and the MEP pathways were induced by SNP, but the MEP pathway was the main contributor to SNP-induced tanshinone production in *S. miltiorrhiza* hairy roots. Accumulation of tanshinone components and expression of *HMGR* and *DXR* were stimulated by water stress. NO probably plays a central role in water stress-induced tanshinone production.

## Abbreviations

ABA: abscisic acid
c-PTIO: 2-(4-Carboxyphenyl)-4,4,5,5-tetramethylimidazoline-1-oxyl-3-oxide
DAD: Photodiode Array Detector
DXR: 1-deoxy-d-xylulose 5-phosphate reductoisomerase
DXS: 1-deoxy-d-xylulose-5-phosphatesynthase
FOS: fosmidomycin
HMGR: 3-hydroxy-3-methylglutaryl coenzyme A reductase
HPLC: high performance liquid chromatography
l-NAME: ***N***^ω^-nitro-l-arginine methyl ester
MVA: mevalonate
MEP: 2-*C*-methyl-d-erythritol-4-phosphate
MEV: mevinolin
NO: Nitric oxide
PEG: polyethylene glycol
SNP: sodium nitroprusside

## References

[1] Dixon R.A. (2001). Natural products and plant disease resistance. Nature.

[2] Zhao J., Davis L.C., Verpoorte R. (2005). Elicitor signal transduction leading to production of plant secondary metabolites. Biotechnol. Adv..

[3] Zhu Z.B., Liang Z.S., Han R.L. (2009). Saikosaponin accumulation and antioxidative protection in drought-stressed Bupleurum chinense DC. plants. Environ. Exp. Bot..

[4] Zhang J., Jia W., Yang J., Ismail A.M. (2006). Role of ABA in integrating plant responses to drought. Field Crops Res..

[5] Mansouri H., Asrar Z., Szopa J. (2009). Effects of ABA on primary terpenoids and Δ^9^-tetrahydrocannabinol in *Cannabis sativa* L. at flowering stage. Plant Growth Regul..

[6] Song L.L., Ding W., Shen J., Zhang Z.G., Bi Y.R., Zhang L.X. (2008). Nitric oxide mediates abscisic acid induced thermotolerance in the calluses from two ecotypes of reed under heat stress. Plant Sci..

[7] Zhang A.Y., Zhang J., Zhang J.H., Ye N.H., Zhang H., Tan M.P., Jiang M.Y. (2011). Nitric Oxide Mediates Brassinosteroid-Induced ABA Biosynthesis Involved in Oxidative Stress Tolerance in Maize Leaves. Plant Cell Physiol..

[8] Huang A.X., She X.P., Cao B., Zhang B., Mu J., Zhang S.J. (2009). Nitric oxide, actin reorganization and vacuoles change are involved in PEG 6000-induced stomatal closure in Vicia faba. Physiol. Plant..

[9] Wang J.W., Zheng L.P., Wu J.Y., Tan R.X. (2006). Involvement of nitric oxide in oxidative burst, phenylalanine ammonia-lyase activation and Taxol production induced by low-energy ultrasound in Taxus yunnanensis cell suspension cultures. Nitric Oxide.

[10] Neill S.J., Desikan R., Clarke A., Hancock J.T. (2002). Nitric Oxide Is a Novel Component of Abscisic Acid Signaling in Stomatal Guard Cells. Plant Physiol..

[11] Zhou L.M., Zuo Z., Chow M.S.S. (2005). Danshen: An overview of its chemistry, pharmacology, pharmacokinetics, and clinical use. J. Clin. Pharmacol..

[12] Yang D., Du X., Liang X., Han R., Liang Z., Liu Y., Liu F., Zhao J. (2012). Different Roles of the Mevalonate and Methylerythritol Phosphate Pathways in Cell Growth and Tanshinone Production of *Salvia miltiorrhiza* Hairy Roots. PLoS ONE.

[13] Bach T.J., Lichtenthaler H.K. (1982). Mevinolin: A highly specific inhibitor of microsomal 3-hydroxy-3-methylglutaryl-coenzyme A reductase of radish plants. Z. Naturforschung.

[14] Kai G., Xu H., Zhou C., Liao P., Xiao J., Luo X., You L., Zhang L. (2011). Metabolic engineering tanshinone biosynthetic pathway in *Salvia miltiorrhiza* hairy root cultures. Metab. Eng..

[15] Liu H.Y., Wang X.D., Wang D.H., Zou Z.R., Liang Z.S. (2011). Effect of drought stress on growth and accumulation of active constituents in *Salvia miltiorrhiza* Bunge. Ind. Crops Prod..

[16] Yang D.F., Ma P.D., Liang X., Wei Z., Liang Z.S., Liu Y., Liu F.H. (2012). PEG and ABA trigger methyl jasmonate accumulation to induce the MEP pathway and increase tanshinone production in *Salvia miltiorrhiza* hairy roots. Physiol. Plant..

[17] Liang Z.S., Yang D.F., Liang X., Zhang Y.J., Liu Y., Liu F.H. (2012). Roles of reactive oxygen species in methyl jasmonate and nitric oxide-induced tanshinone production in *Salvia miltiorrhiza* hairy roots. Plant. Cell Rep..

[18] Dai Z.B., Cui G.H., Zhou S.F., Zhang X.A., Huang L.Q. (2011). Cloning and characterization of a novel 3-hydroxy-3-methylglutaryl coenzyme A reductase gene from *Salvia miltiorrhiza* involved in diterpenoid tanshinone accumulation. J. Plant. Physiol..

[19] Yan X., Zhang L., Wang J., Liao P., Zhang Y., Zhang R., Kai G. (2009). Molecular characterization and expression of 1-deoxy-d-xylulose 5-phosphate reductoisomerase (DXR) gene from *Salvia miltiorrhiza*. Acta Physiol. Plant..

[20] Wu S.J., Shi M., Wu J.Y. (2009). Cloning and characterization of the 1-deoxy-d-xylulose 5-phosphate reductoisomerase gene for diterpenoid tanshinone biosynthesis in *Salvia miltiorrhiza* (Chinese sage) hairy roots. Biotechnol. Appl. Biochem..

[21] Zhao M.G., Tian Q.T., Zhang W.H. (2007). Nitric oxide synthase-dependent nitric oxide production is associated with salt tolerance in Arabidopsis. Plant. Physiol..

[22] Foissner I., Wendehenne D., Langebartels C., Durner J. (2000). *In vivo* imaging of an elicitor-induced nitric oxide burst in tobacco. Plant. J..

[23] Wang J.W., Wu J.Y. (2005). Nitric oxide is involved in methyl jasmonate-induced defense responses and secondary metabolism activities of Taxus cells. Plant. Cell Physiol..

[24] Xu M.J., Dong J.F. (2005). Nitric oxide stimulates indole alkaloid production in Catharanthus roseus cell suspension cultures through a protein kinase-dependent signal pathway. Enzym. Microb. Technol..

[25] Zhao Z.G., Chen G.C., Zhang C.L. (2001). Interaction between reactive oxygen species and nitric oxide in drought-induced abscisic acid synthesis in root tips of wheat seedlings. Aust. J. Plant. Physiol..

[26] Yang D., Sheng D., Duan Q., Liang X., Liang Z., Liu Y. (2012). PEG and ABA Trigger the Burst of Reactive Oxygen Species to Increase Tanshinone Production in *Salvia miltiorrhiza* Hairy Roots. J. Plant. Growth Regul..

[27] Hagen C., Grunewald K. (2000). Fosmidomycin as an inhibitor of the non-mevalonate terpenoid pathway depresses synthesis of secondary carotenoids in flagellates of the green alga Haematococcus pluvialis. J. Appl. Bot..

